# Fasting as a Therapy in Neurological Disease

**DOI:** 10.3390/nu11102501

**Published:** 2019-10-17

**Authors:** Matthew C.L. Phillips

**Affiliations:** Department of Neurology, Waikato Hospital, Hamilton 3204, New Zealand; Matthew.Phillips@waikatodhb.health.nz

**Keywords:** fasting, therapy, neurological disease, metabolic syndrome, cancer, neurodegeneration, stroke, epilepsy, multiple sclerosis

## Abstract

Fasting is deeply entrenched in evolution, yet its potential applications to today’s most common, disabling neurological diseases remain relatively unexplored. Fasting induces an altered metabolic state that optimizes neuron bioenergetics, plasticity, and resilience in a way that may counteract a broad array of neurological disorders. In both animals and humans, fasting prevents and treats the metabolic syndrome, a major risk factor for many neurological diseases. In animals, fasting probably prevents the formation of tumors, possibly treats established tumors, and improves tumor responses to chemotherapy. In human cancers, including cancers that involve the brain, fasting ameliorates chemotherapy-related adverse effects and may protect normal cells from chemotherapy. Fasting improves cognition, stalls age-related cognitive decline, usually slows neurodegeneration, reduces brain damage and enhances functional recovery after stroke, and mitigates the pathological and clinical features of epilepsy and multiple sclerosis in animal models. Primarily due to a lack of research, the evidence supporting fasting as a treatment in human neurological disorders, including neurodegeneration, stroke, epilepsy, and multiple sclerosis, is indirect or non-existent. Given the strength of the animal evidence, many exciting discoveries may lie ahead, awaiting future investigations into the viability of fasting as a therapy in neurological disease.

## 1. Introduction

Fasting has surged in popularity over the new millennium. Much of its newfound enthusiasm has been driven by a growing public perception that fasting may be beneficial for many aspects of human health. Despite the purported health benefits of fasting, it remains somewhat foreign to conventional medical practice, although this situation is not exactly new; fasting has historically fallen in and out of fashion in its relationship to medicine. As Mark Twain may have said, “History does not repeat itself, but it rhymes.”

To understand why and how fasting may be applicable as a therapy to an array of neurological diseases, it is helpful to examine fasting in both evolutionary and mechanistic contexts. In doing so, it should gradually become apparent that fasting and medication-based approaches need not be mutually exclusive; they can be combined, and such an approach may actually be ideal. In an era of rising healthcare costs and an increasing prevalence of disabling neurological disorders, the impact of a self-empowering, cost-free, effective therapy alongside conventional medical approaches would be substantial and positive.

On this background, the definition, origins, mechanisms, and various regimens of fasting are discussed, followed by a summary of the evidence supporting fasting in the prevention and treatment of a variety of neurological disorders, followed lastly by a discourse on the most common adverse effects and misconceptions associated with fasting.

## 2. What Is Fasting?

“Fasting” may be defined as a voluntary abstinence from food and drink for specified, recurring periods of time, with the fasting periods typically ranging from 12 hours to three weeks in humans [1,2,3,4]. Fasting is most often contrasted with ad libitum (“as desired”) feeding, which is characterized by three or more meals per day in modern societies, and—combined with a sedentary lifestyle—may increase a person’s risk of developing a chronic neurological disease [5]. Fasting should not be confused with starvation, a state of chronic nutritional insufficiency which is neither voluntary nor controlled, and which may culminate in organ failure and death.

### 2.1. Fasting: Origins

In evolution, organisms able to tolerate environments devoid of nutrients for extended periods of time held an important survival advantage over those unable to do so. The evolutionary selection pressure to survive the stresses associated with low-energy environments has produced a number of fasting-induced metabolic mechanisms that have been conserved for millions, if not billions, of years in humans [6].

#### 2.1.1. Pre-Human Evolutionary Origins of Fasting

Many single-celled and simple multicellular organisms alter their metabolism during times of nutrient scarcity, the aim of which is to conserve resources, minimize damage, and enhance longevity. For example, when mutant *Escherichia coli* bacteria are transferred from a nutrient-rich broth to a calorie-free medium, they undergo a series of metabolic changes that allow them to survive four times longer than wild-type bacteria [7], and when the yeast *Saccharomyces cerevisiae* is swapped from a growth medium to water, it enters a stationary phase that increases its stress tolerance and doubles its lifespan [8,9]. Similar responses have also been observed in simple multicellular organisms deprived of nutrients, such as the nematode *Caenorhabditis elegans*, which transitions to a metabolic “dauer state,” resulting in a substantial increase in lifespan [10].

Beyond these simpler lifeforms, a number of complex multicellular organisms, such as lungfish, eels, frogs, snakes, and arthropods, have also evolved extraordinary resistances to nutrient scarcity, partly due to decreased resting metabolic rates and activity levels [11]. However, rather than enter a dormant phase, some complex organisms actually increase their cognitive and physical activity levels when fasted, improving their ability to seek and acquire food. Rodents on a fasting regimen, for example, have shown decreases in the size of most organs, aside from the brain (and gonads) [12], resulting in maintained or improved cognitive and physical performance [13,14]. In another example, captive lions switched from a conventional daily feeding schedule to a “gorge and fast” schedule consisting of only three meals per week have shown a reduction in maladaptive, stereotypic behaviors, such as pacing, and an increase in adaptive, hunting-related behaviors, such as sniffing and stalking [15].

#### 2.1.2. Fasting in Human History

Like many of the organisms that preceded them in evolution, pre-agricultural humans endured regular periods of food scarcity [16]. Humans have been hunter-gatherers for two million years; it was only a relatively short 12,000 years ago that the transition to agriculture occurred [17]. Thus, post-agricultural humans may not have had sufficient time to fully adapt to the continuous food supply provided by farming, which may in part explain the later introduction of voluntary fasting practices by the majority of civilizations on earth [18]. The ancient Romans, for example, believed that eating more than one large meal per day was unhealthy [19]. Most world religions, including Christianity and Islam, also incorporated regular fasting into their religious practices [20].

In more modern times, the potential health benefits of fasting have been intermittently recognized—and forgotten. The American physician Edward Dewey adopted a somewhat radical view of fasting in the 1800s, believing that virtually all disease stemmed from excessive eating [21]. In the 1900s, German physician Otto Buchinger, the first person to rigorously document the beneficial effects of fasting in many human diseases, wrote that “Fasting is, without any doubt, the most effective biological method of treatment” [22]. Valter Longo, an Italian-born biogerontologist and fasting researcher in the 2000s, has recently suggested that fasting selectively activates multiple “longevity programs” which may lead not only to an extended lifespan, but also to an extended healthspan [23]. Curiously, despite these and other fasting advocates, the established eating pattern in most modern societies remains three or more meals per day, a pattern that is associated with a globally increasing prevalence of obesity, type 2 diabetes, and a variety of disabling neurological disorders [24,25].

### 2.2. Fasting: Mechanisms

**Figure 1:**
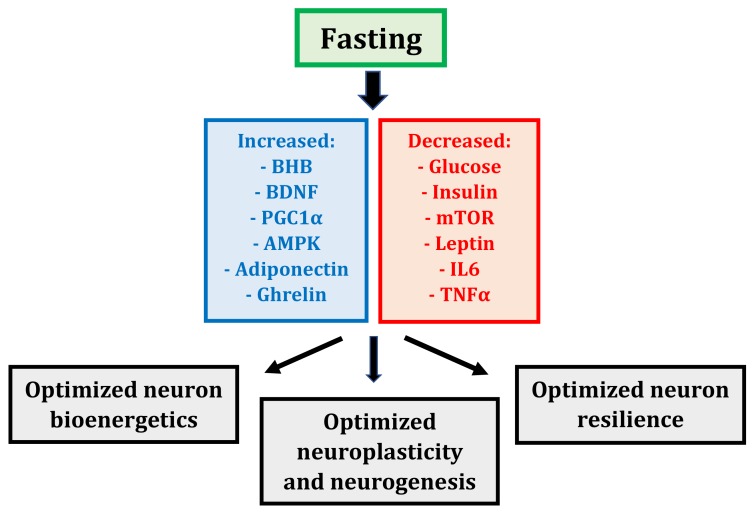
Fasting-induced metabolic and transcriptional mechanisms and their effects on neurons (BHB—beta-hydroxybutyrate; BDNF—brain-derived neurotrophic factor; PGC1α—peroxisome proliferator-activated receptor γ coactivator 1α; AMPK—AMP-activated protein kinase; mTOR—mammalian target of rapamycin; IL6—interleukin 6; TNFα—tumor necrosis factor α).

Fasting induces the coordinated alteration of many metabolic and transcriptional mechanisms that may influence neurons (Figure 1). Collectively, these alterations produce a whole-body, altered metabolic state that optimizes neuron bioenergetics, plasticity, and resilience to stress, culminating in maintained—or even enhanced—cognitive performance [5].

#### 2.2.1. Fasting: A Whole-Body, Altered Metabolic State

Following 12–36 hours of fasting, the human body enters a physiological state of ketosis characterized by low blood glucose levels, exhausted liver glycogen stores, and the hepatic production of fat-derived ketone bodies, or ketones, which serve as a major energy source for the brain [26]. The liver is the primary site of ketogenesis, but brain astrocytes also generate ketones [27,28]. Within several days of initiating a fast, ketones become the brain’s preferred fuel source, providing up to 70% of its energy requirements [29]. Ketones constitute a more efficient source of energy per unit oxygen in muscles [30,31], and possibly in the brain [32], enhancing neuron bioenergetics and cognitive performance; for example, it has been shown that rodents subjected to a ketone ester for five days exhibit improved spatial learning and memory [33].

Yet ketones are more than just an energy source for neurons; the primary blood ketone, beta-hydroxybutyrate (BHB), also serves important signaling functions [5,26]. In hippocampal and cortical neurons, BHB plays a vital signaling role by inducing the transcription of brain-derived neurotrophic factor (BDNF) via its inhibition of histone deacetylases, enzymes that repress BDNF expression [34]. BDNF is a pivotal regulator of neuron function; it stimulates mitochondria biogenesis, maintains synaptic structure, spurs the production and survival of new hippocampal neurons, and enhances neuron resistance to injury and disease [35].

In addition to BHB and BDNF, fasting induces the expression of a master regulator of mitochondria, the transcription factor peroxisome proliferator-activated receptor γ coactivator 1α (PGC1α) [5,36]. PGC1α is a central inducer of mitochondria biogenesis, increasing mitochondria biomass, which in turn enhances neuron bioenergetics and enables synaptic plasticity. PGC1α also modulates the composition and function of mitochondria; for example, muscle mitochondria isolated from transgenic mice that ectopically express PGC1α exhibit an increased respiratory capacity compared to wild-type controls [37]. Thus, PGC1α not only stimulates mitochondria biogenesis, it also stimulates the formation of mitochondria with altered intrinsic properties; both have a positive effect on neuron bioenergetics.

Fasting displays potent effects on glucose metabolism and insulin signaling [1,6]. In humans, fasting for three-to-five days decreases blood glucose levels by 30%–40%, and inhibits glycolysis [38,39,40]. Fasting on alternate days for three weeks decreases insulin levels by 50%–60% on the fasted day [41]. In general, three-to-five days of fasting in humans also results in a 60% decline in insulin-like growth factor (IGF-1), the chief growth factor in mammals, a five-to-ten-fold increase in IGF-1 binding protein (IGFBP1), one of its main binding proteins, and a two-to-three-fold increase in growth hormone (GH), which rises to preserve muscle mass [39,42,43]. Fasting therefore prevents the development of chronic, excessive, and potentially dysregulated glucose metabolism while concurrently preserving insulin sensitivity and growth factor signaling, all of which may benefit neuron bioenergetics.

Fasting also exerts a powerful influence cell synthesis and degradation processes [44,45]. The balance of cell synthesis versus degradation is regulated by the respective activities of two master regulators of metabolism, mammalian target of rapamycin (mTOR) and AMP-activated protein kinase (AMPK) [44]. Under high-nutrient conditions (particularly amino acids), mTOR stimulates protein synthesis and cell growth; in contrast, when cell energy reserves are low, AMPK downregulates mTOR to minimize energy consumption and stimulate autophagy, an intracellular degradation pathway that clears misfolded proteins and damaged organelles, recycles nutrients, and bolsters energy production [45]. Fasting suppresses mTOR and elevates AMPK, thereby limiting nutrient consumption and growth in favor of autophagy and survival; although mTOR and AMPK have mostly been studied in muscle cells, recent evidence suggests these two antagonistic master metabolic regulators may also mediate fasting responses in neurons [5].

Fasting influences fat metabolism by altering the hormonal activities of leptin, adiponectin, and ghrelin [1,5,46]. Leptin is associated with a pro-inflammatory state, whereas adiponectin is associated with enhanced insulin sensitivity and suppressed inflammation [47]. Ghrelin is also associated with enhanced insulin sensitivity [48]; moreover, ghrelin may stimulate hippocampal synaptic plasticity and neurogenesis [49]. Fasting decreases leptin but increases adiponectin and ghrelin, alterations that are probably beneficial for neuron bioenergetics and the maintenance of neural pathways.

Lastly, fasting suppresses inflammation, reducing the expression of pro-inflammatory cytokines such as interleukin 6 (IL6) and tumor necrosis factor α (TNFα) [50,51]. Since inflammatory processes underpin many different neurological disorders [52], the ability of fasting to suppress neural and systemic inflammation may improve neuron survival in these disorders.

#### 2.2.2. Fasting: More Than Just Calorie Restriction

Calorie restriction refers to a chronic 20%–40% reduction in calorie intake, with meal frequency maintained [1]. Over a century of research has shown that calorie restriction reduces chronic disease and lengthens lifespan in a variety of species [53]. Since calorie restriction and fasting share many similar mechanisms and fasting often produces a decrease in calorie intake over time, the question is often raised as to whether the potential benefits of fasting are merely due to reduced calorie intake, as opposed to any particular effect of the fasting. 

Several studies in animals and humans have indicated that fasting may confer benefits on cell (including neuron) metabolism beyond calorie restriction. It has been shown that some mice fasted on alternate days can eat twice as much on the feeding day, such that their net weekly calorie intake remains similar to mice fed ad libitum; despite the lack of overall calorie restriction, the former still display beneficial metabolic effects compared to the latter, including improved glucose levels and insulin activity, as well as enhanced neuron resistance to a neurotoxin, kainic acid [54]. Studies involving overweight and obese non-diabetic humans have shown greater improvements in insulin sensitivity in fasted individuals compared to their non-fasted, calorie-matched counterparts [55,56]. Recently, a five-week randomized crossover trial in men with pre-diabetes compared a fasting regimen, containing sufficient overall calorie intake to prevent weight loss, against a control group with a regular eating schedule; although both groups were matched for calorie intake, the fasting group exhibited greater improvements in insulin sensitivity and other measures of metabolic health [57].

The most obvious explanation for a putative, fasting-specific effect on metabolic health may lie in the fundamental distinction between fasting and calorie restriction—timing. Specifically, fasting is applied intermittently, whereas calorie restriction is continuous. Following 12–36 hours of fasting, there is a discernible metabolic transition or “switch” from utilizing carbohydrates and glucose to fatty acids and ketones as the major cellular fuel sources [5]. During the fasted state, the switch is “on,” theoretically upregulating autophagy and survival pathways in neurons, whereas during the fed state, the switch is “off,” emphasizing remodeling and growth pathways. Thus, unlike calorie restriction, fasting capitalizes on each sequential bioenergetic challenge by “setting the stage” for a relatively stress-free cell recovery phase; in other words, it is the switching—the intermittency—that may provide the advantage for neuron metabolism. Indeed, chronicity can be harmful, regardless of a fed or fasted metabolic state—for example, acute mTOR activation promotes muscle hypertrophy, whereas chronic activation produces atrophy [58,59,60], and intermittent AMPK activation enhances neuroplasticity, but sustained AMPK activation impairs it [61]. 

### 2.3. Fasting: Regimens

Three parameters characterize a fasting regimen—the intensity of the food and drink restriction, the frequency of the fasting periods, and the duration of the fasting periods (Table 1). The “ideal” fasting regimen depends on individual lifestyle and tolerability.

#### 2.3.1. Intensity of the Food and Drink Restriction

The “intensity” of a fast refers to the amount and type of food and drink that may be permitted during the fasting periods. The intensity of a fasting period ranges from the complete omission of all food and drink (a “pure” fast) to a minimal intake of specific meals aimed at maintaining the fasted metabolic state.

Fasts that eliminate all food and drink are conceptually simple, but from a practical standpoint, the lack of water intake imposes a realistic maximum upper limit of 24 hours. An example of this type of fast occurs during the Islamic month of Ramadan, in which individuals abstain from all food and drink from sunrise to sunset, for approximately 30 days [2,62].

Water-only fasts omit all calorie intake but provide adequate hydration and can therefore be extended out to several days, weeks, or even months, provided that adequate salt and other micronutrients are maintained [63,64]. Fluid-only fasts additionally permit calorie-free fluids, such as tea and black coffee, which can help maintain energy and suppress the transient waves of hunger that may occur in some people. Both types of fasts should aim for a minimum of 2–2.5 L of water or fluid intake per day [65], and a multivitamin may be added to provide micronutrients [66]. 

For individuals who have difficulty tolerating fluid-only fasts, a degree of fasting intensity can be exchanged for improved tolerability using specific meal choices that do not disrupt the fasted metabolic state. The caloric intake of these meals should not exceed 250–500 kcal per day [65]. One common option is to incorporate a daily vegetable or bone broth into the fast, which also provides fluids and micronutrients [65,66].

#### 2.3.2. Frequency and Duration of the Fasting Periods

Most strains of mice cannot survive for more than three days without food, but most humans can survive fasting periods of 30 days or longer [1]. Given this ability to fast for extended time periods, substantial variability exists in the frequency and duration of fasting regimens available to humans [2,3,4,67].

The most tolerable of all fasting regimens may be time-restricted feeding (TRF), which consists of daily fasting periods lasting 12–20 hours, alternating with a daily four-to-twelve hour “eating window” [2,3,4,67,68]. There is some evidence that restricting the eating window to the morning or middle of the day produces superior effects on body fat and insulin resistance compared to late afternoon or evening eating windows [68].

Fasting periods lasting longer than a day are often grouped under the broadly-used term “intermittent fasting,” the definition of which often varies depending on the source [2,3,4,67]. In a practical sense, it is probably best to use reserve the use of this term for fasting regimens containing recurring fasting periods lasting 24–48 hours in duration. In human studies, the most common intermittent fasting regimens are alternate-daily fasting (ADF) and fasting for two consecutive days per week (two-days-per-week fasting).

Periodic fasting typically refers to extended fasting periods lasting from two days to three weeks in duration [3]. Periodic fasting may produce more pronounced metabolic changes compared to TRF, ADF, or two-days-per-week fasting; however, for many people, periodic fasting is difficult to tolerate and may not be necessary, depending on an individual’s goals. Fasting periods lasting several months to over a year have been documented in humans [64,69,70], but these represent exceptional cases.

## 3. Evidence Supporting Fasting in Neurological Disease

Fasting may delay aging, a major risk factor for neurological disease [1,3,4]. Beyond aging, compelling evidence in animals and humans has indicated that fasting can prevent and treat the metabolic syndrome, another major risk factor for a variety of neurological diseases [71]. Fasting can also prevent and treat many neurological disorders in animals; due to a lack of research, much less evidence is available in humans (Table 2). More human studies are needed.

### 3.1. Metabolic Syndrome

The metabolic syndrome consists of a combination of abdominal obesity, insulin resistance, hypertension, and dyslipidemia [72]. Fasting alleviates the key abnormalities of the metabolic syndrome in animals and humans, resulting in beneficial effects that are similar—and sometimes superior—to those observed with calorie restriction.

#### 3.1.1. Fasting as a Therapy in the Metabolic Syndrome: Animal Studies

Fasting consistently mitigates obesity in animal models [1,73]. Rodents maintained on fasting regimens exhibit lower body weights compared to rodents fed ad libitum, with long-term (over 20 weeks) fasting regimens generally producing significant weight loss [54,74,75,76]. Moreover, the lower body weights largely result from reduced fat mass, not lean mass; the latter is spared [77,78].

In animals, fasting regimens also eradicate visceral fat and improve insulin sensitivity [3,4]. Fasting cures type 2 diabetes in rodent models, an effect that is not due to calorie restriction given that TRF animals consume the same overall calories as animals fed ad libitum, yet the former display an anti-diabetic effect whereas the latter do not [79,80]. In some studies, fasting regimens have induced beneficial effects on insulin resistance that are superior to those induced by even severe calorie restriction; for example, rodents on ADF can maintain similar body weights compared to rodents fed ad libitum, yet the former still show improvements in glucose levels and insulin activity that are as great as, or greater than, those demonstrated by rodents on a 40% calorie restriction [54].

ADF decreases heart rate and blood pressure in rodents within days, with both continuing to decrease until stabilizing at lower levels by the end of a month, after which they remain low on both fasting and feeding days [75]. Rodents on an ADF regimen also show increases in heart rate variability [81], as well as superior cardiovascular adaptation to an immobilization stress [75]. The fasting-mediated effects on heart rate, blood pressure, heart rate variability, and cardiovascular stress adaptation are thought to result from increases in BDNF, which enhances the cholinergic activity of brainstem cardiovagal neurons [3]. They do not appear to be mediated by calorie restriction, given that rodents on ADF, with an overall 10%–20% calorie restriction, show greater decreases in resting heart rate than rodents maintained on a chronic 40% calorie restriction [81].

#### 3.1.2. Fasting as a Therapy in the Metabolic Syndrome: Human Studies

The evidence for fasting-induced weight loss in humans is not as consistent as in animals [73]; however, short-term (under six months) fasting regimens generally lead to weight loss in overweight and obese individuals [2,3,82]. Many people undergoing religious fasts experience weight loss, but it is often regained afterwards [83,84]. Conversely, reviews of overweight and obese people placed on two-to-six month fasting regimens generally demonstrate a 3%–16% reduction in body weight compared to controls, with regimens over three months more likely to show a clinically meaningful weight reduction of 5 kg or more [2,4,85]. Both fasting and calorie restriction regimens produce similar degrees of weight loss, although some studies suggest that fasting regimens may be superior to a 20%–25% calorie restriction [56,86]. Moreover, despite similar decreases in body weight, fasting may be more effective than calorie restriction at retaining lean mass [85].

Fasting has been known to reverse type 2 diabetes in humans for well over a century, often eliminating the need for diabetic medications [66,87,88,89]. Moreover, fasting regimens appear to exert insulin-sensitizing effects independent of weight loss [90,91], and non-diabetic and pre-diabetic individuals undergoing fasting regimens show greater improvements in insulin sensitivity compared to non-fasted individuals matched for calorie intake [55,56,57]. Collectively, these findings suggest that the insulin-sensitizing effects of fasting are, at least to some extent, independent of weight loss and calorie restriction.

In humans, six-to-twenty-four weeks of ADF or two-days-per-week fasting induces a significant decrease in blood pressure (3%–8% systolic and 6%–10% diastolic), generally in the context of weight loss [55,92,93]. Periodic fasting is particularly effective at reducing blood pressure in hypertensive individuals; systolic blood pressure falls by 20–60 mmHg within one-to-two weeks [94,95]. Fasting and calorie restriction show similar effects on blood pressure reduction [55,56].

### 3.2. Cancer

Cancer cells exhibit many metabolic alterations, most notably a substantially elevated rate of glycolysis despite the presence of normal oxygen concentrations, known as the “Warburg effect” [96,97]. The Warburg effect is inefficient at producing energy; hence, it relies upon a dramatically increased rate of glucose uptake by cancer cells, a feature common to over 90% of malignant cancers [98]. In addition to glucose, some cancer cells are highly reliant on the amino acid glutamine for their growth and proliferation [99,100]. The heavy dependence of cancer cells on glucose and glutamine is thought to result from deficiencies in mitochondria number, structure, and function that are characteristic of most cancers [101], as well as a “reprogrammed” cell metabolism supportive of unchecked growth and proliferation [97]. In addition to these metabolic hallmarks, cancer cells exhibit deregulated, hyperactive insulin, IGF-1, and mTOR signaling [58,102,103], as well as dysfunctional autophagy [45]. 

#### 3.2.1. Fasting as a Therapy in Cancer: Animal Studies

In 1914, Rous reported that a reduction in food intake decreased the incidence of cancer in rodents [104]. Since then, a number of studies have collectively shown that calorie restriction regimens reduce tumor incidence by perhaps 75% in rodents [105] and by 50% in rhesus monkeys [106]. The explanation for these findings may partly relate to the fact that calorie restriction reduces blood glucose and growth factor availability, dampening cancer cell growth [107]. Fasting regimens also prevent tumors in most rodent cancer models; however, the results are more variable in comparison with calorie restriction [105]. These findings might be explained by variability across studies with regards to the specific fasting regimen used, as well as the macronutrient ratio employed in the refeeding periods; both may strongly influence the degree of tumor prevention, which in many cases exceeds the tumor-preventive effects of calorie restriction [108,109,110,111]. Compared to calorie restriction, fasting results in the production of large amounts of ketones, which cannot be effectively utilized by cancer cells and may inhibit their growth [112,113,114]. Fasting also intermittently reduces glucose, glutamine, and growth factor availability to a greater extent than calorie restriction, which theoretically deprives cancer cells of their major fuels and disrupts their ability to proliferate.

Beyond prevention, there is some evidence that fasting can treat established tumors in animals. Rodents intraperitoneally inoculated with tumor cells, for example, display a 50% survival rate after 10 days of ADF, compared to only 12.5% survival with ad libitum feeding [115]. Moreover, substantial evidence has shown that fasting works in concert with chemotherapy by creating a cellular state of “differential stress resistance” whereby energy-deprived normal cells prioritize energy conservation and survival by activating stress resistance pathways, becoming more resistant to the extreme conditions created by limited nutrient availability and chemotherapy [45,107]. In contrast, energy-deprived cancer cells continue to emphasize growth and proliferation; since they do not activate stress resistance pathways, they become vulnerable to the stresses imposed by limited nutrient availability and chemotherapy. In support of the concept of differential stress resistance, fasting has been shown to improve the therapeutic responses of a variety of rodent cancer models, including gliomas, to chemotherapy [45,116].

#### 3.2.2. Fasting as a Therapy in Cancer: Human Studies

There are little data regarding the effects of fasting in human cancer prevention. As in animals, fasting fosters a nutrient-deprived environment that may be hostile to cancer cells [107]. In terms of specific evidence, however, there have only been observational studies, which can only be suggestive; in a population of 2337 breast cancer survivors, for example, shorter nightly fasts were associated with an increased recurrence of cancer [117].

Several small studies have shown that that fasting may hold promise in treating established cancers in humans, including primary brain tumors as well as cancers that may metastasize to the brain. Glioblastomas, the most common primary malignant brain tumors in adults, have a median survival time of one-to-two years [118]. Case reports involving glioblastoma patients using water-only fasting regimens in conjunction with other forms of cancer treatment have reported favorable outcomes with respect to tumor growth [119,120]. The potential utility of fasting alongside chemotherapy has also been assessed in other cancers, including those that often metastasize to the brain, such as lung and breast cancer. In a case series involving ten individuals, including one with lung cancer and four with breast cancer, fasting before or after chemotherapy decreased chemotherapy-related adverse effects, such as weakness, fatigue, and gastrointestinal upset [121]. Moreover, a randomized trial involving 13 women with breast cancer, randomized to either 24 hours of fasting before and after chemotherapy or ad libitum feeding, demonstrated that fasting was well-tolerated, prevented chemotherapy-induced decreases in red blood cell and platelet counts, and possibly protected normal cells from DNA damage [122]. Furthermore, in a case series involving 20 patients, including one with lung cancer and five with breast cancer, fasting for 48 hours or longer before and during chemotherapy modestly protected normal cells from DNA damage [123].

### 3.3. Neurodegeneration

Neurodegenerative disorders, such as Huntington’s disease (HD), Parkinson’s disease (PD), and Alzheimer’s disease (AD), afflict different neurons (striatal spiny neurons in HD, widespread dopaminergic and cholinergic neurons in PD, and cortical cholinergic neurons in AD); however, all three disorders exhibit impaired neuron bioenergetics, glucose metabolism, and neurotrophic factor signaling [3,124]. In all three, there is a reduced expression of the master mitochondria regulator PGC1α, along with an associated decline in mitochondria biogenesis and function [36,124]. Moreover, the respiratory chain is impaired in PD and AD, especially PD, which demonstrates a marked deficit at complex I [125]. Furthermore, both PD and AD show impairments in neuron glucose metabolism and insulin signaling [126,127], especially AD, which is characterized by brain insulin deficiency as well as resistance, thus leading to AD being described as a form of brain-specific, “type 3” diabetes [128]. 

#### 3.3.1. Fasting as a Therapy in Neurodegeneration: Animal Studies

Fasting improves cognition and prevents cognitive decline in non-neurodegenerative animal models. Rodents on fasting regimens display enhanced cognitive performance compared to those fed ad libitum [14,129]. TRF stalls age-related declines in brain white matter integrity, energy production, and cognition observed in rodents fed ad libitum [130,131]. Mice maintained on TRF also show increased hippocampal BDNF levels, synaptic strength, and neurogenesis [132,133], suggesting that the improvements in cognition are, to some extent, mediated by BDNF.

Fasting usually slows neurodegeneration in animal models of HD, PD, and AD [1]. Huntington mutant mice show deficiencies in striatal and cortical BDNF levels as well as glucose metabolism, followed by neurodegeneration and motor dysfunction; however, if ADF is commenced early enough, BDNF levels increase, glucose metabolism normalizes, and neurodegeneration and motor dysfunction are delayed [134]. In PD mouse models, ADF confers protection against the dopaminergic neuron degeneration and loss induced by the mitochondria toxin 1-methyl-4-phenyl-1,2,3,6-tetrahydropyridine (MPTP), resulting in improved functional outcomes compared to mice fed ad libitum [135]. Since MPTP interferes with complex I of the mitochondria respiratory chain, this beneficial effect may partly be due to the ketones produced by fasting, which theoretically circumvent the PD complex I defect via a complex II-dependent mechanism, enhancing mitochondria bioenergetics [136]. In AD mouse models, ADF has been shown to confer increased hippocampal neuron resistance to the neurotoxic effects of kainic acid, resulting in improved cognitive performance [54,137], and ADF ameliorates age-related cognitive deficits that occur in transgenic mice expressing beta-amyloid precursor protein, presenilin 1, and tau mutations [138].

#### 3.3.2. Fasting as a Therapy in Neurodegeneration: Human Studies

To date, fasting has not been explored as a therapy in people with HD, PD, and AD. However, indirect evidence has been provided by studies of ketogenic diets in these disorders [139]. Ketogenic diets are high-fat, adequate-protein, low-carbohydrate diets that force the body to burn fats rather than carbohydrates as the primary energy source, thus mimicking a fasted metabolic state by generating ketones and inducing many of the metabolic mechanisms induced by fasting. In PD, a small case series showed improved motor symptoms after four weeks of a ketogenic diet [140], and a subsequent randomized controlled study involving 47 people with mild-to-severe PD showed improvements in many of the most disabling, least levodopa-responsive PD nonmotor symptoms after eight weeks of a ketogenic diet [141]. Regarding the effects of a ketogenic diet in AD, a single case series involving 15 people with mild-to-moderate AD reported mild improvements in cognition after 12 weeks of such a diet [142]; these findings may be partly explained by the fact that although brain glucose uptake is markedly impaired in AD, ketone utilization is not [143].

### 3.4. Stroke

A stroke is a neurological deficit of sudden onset due to an interrupted blood supply, resulting in brain, spinal cord, or retinal infarction [144]. Most strokes worldwide are ischemic and involve neuron loss, neuroinflammation, neural network rewiring, and neuron functional reorganization. 

#### 3.4.1. Fasting as a Therapy in Stroke: Animal Studies

In animals, fasting prior to an ischemic stroke alleviates brain damage and enhances functional recovery. Rodents maintained on ADF prior to occlusion of the middle cerebral artery display less brain damage and improved functional outcomes compared to those fed ad libitum [50,145]. Moreover, mice maintained on TRF for three months prior to middle cerebral artery occlusion show increased neurogenesis in the hippocampus and subventricular zones, as well as infarcts less than half the size of those seen in mice fed ad libitum [146]. Furthermore, rats maintained on TRF for three months before and 70 days after global cerebral ischemia show persistent improvements in spatial memory compared to non-fasting controls [147]. The effects of fasting after an ischemic stroke has already occurred are not known, although indirect evidence is available from traumatic brain and spinal cord studies which demonstrate that the implementation of a fasting regimen after a traumatic brain injury confers neuroprotection and improves functional recovery [148,149]. Previously, it has been shown that damaged rat cortex exhibits a striking, 8.5-fold increase in BHB uptake compared to sham animals [150], which suggests that much of the fasting-mediated recovery in ischemic stroke may be due to the increased metabolic efficiency of BHB compared to glucose. However, it is likely that upregulated BDNF, enhanced mitochondria function, activated stress response signaling pathways, and suppressed neuroinflammation also play important roles [5].

#### 3.4.2. Fasting as a Therapy in Stroke: Human Studies

Human studies on the direct effects of fasting in ischemic stroke are lacking. However, fasting reduces levels of pro-inflammatory factors, such as C-reactive protein, IL6, and homocysteine [51], which may inhibit the formation of atherosclerotic plaques, a common source of stroke in humans.

### 3.5. Epilepsy

Epilepsy is characterized by neuron hyperexcitability, leading to an enduring predisposition to generate seizures [151]. Despite an array of anti-epileptic drugs and the availability of surgery, one-third of people with epilepsy continue to experience drug-resistant seizures. 

#### 3.5.1. Fasting as a Therapy in Epilepsy: Animal Studies

Modest evidence supports fasting for seizure control in animal models of epilepsy. Compared to mice fed ad libitum, mice on TRF show a prolonged latency to seizure generation and a decrease in the severity and frequency of seizures [152]. Such an anti-seizure effect is at least partly due to the direct anticonvulsant effects of BHB [153,154]. However, fasting may additionally confer seizure protection by altering the activities of metabolic factors such as IGF-1, mTOR, and AMPK.

#### 3.5.2. Fasting as a Therapy in Epilepsy: Human Studies

Fasting has been used to treat epilepsy since the era of Hippocrates [155], but it was not until 1911 that Guelpa and Marie formally documented the effectiveness of fasting in the treatment of 20 people with epilepsy [156]. With the introduction of Wilder’s ketogenic diet and a long succession of anti-epileptic drugs, virtually no studies of fasting in epilepsy were published for nearly a century. Recently, a small study investigated the effects of a two-month modified TRF regimen in six epileptic children with an incomplete response to a ketogenic diet, reporting that four of the six children experienced modest improvements in seizure control [157]. These results are not surprising, as fasting and ketogenic diets share many similar mechanisms; for example, both increase BHB, which in some studies has correlated with improved seizure control [158,159], and both induce additional mechanisms that collectively stabilize synaptic function. However, since some of the fasted children experienced modest improvements in seizure control beyond those of a ketogenic diet, there may be important differences in the anti-seizure mechanisms underlying fasting and ketogenic diets.

### 3.6. Multiple Sclerosis

Multiple sclerosis (MS) is an inflammatory, autoimmune-mediated disorder that damages central nervous system neurons and their axons [160]. Recently, there has been an increasing focus on the role of gut bacteria and their metabolites in MS, given that both are important regulators of T cell differentiation and enteric immune responses. This suggests that dietary factors, which exert a strong influence on gut microbiota composition and metabolite production, may contribute to the pathogenesis of MS [161].

#### 3.6.1. Fasting as a Therapy in Multiple Sclerosis: Animal Studies

Fasting is beneficial in experimental autoimmune encephalomyelitis (EAE), an animal model of MS that involves the inflammatory-mediated demyelination and death of oligodendrocytes [162]. In mice, ADF ameliorates the pathological and clinical features of EAE, enhances gut bacteria diversity, and increases regulatory T cell numbers [163]. Moreover, fecal microbiota transfers from ADF mice to mice fed ad libitum decrease the severity of EAE in the latter, indicating that some of the benefits of fasting may be mediated by gut bacteria [163]. Alternating cycles of a fasting-mimicking diet (FMD), which mimics fasting by providing a standard amount of food severely reduced in calorie density, also reduce the clinical severity of EAE in mice, including a complete reversal of symptoms in 20% of them [164]. Potential mechanisms underlying the FMD in EAE include enhanced oligodendrocyte precursor cell regeneration and axon remyelination, as well as improved regulation of autoimmune cells and pro-inflammatory markers.

#### 3.6.2. Fasting as a Therapy in Multiple Sclerosis: Human Studies

Fasting holds promise as a therapy in human inflammatory-mediated diseases, although there is no direct evidence supporting it as a therapy in MS. Fasting produces pathological and clinical improvements in non-neurological, inflammatory-mediated diseases, such as rheumatoid arthritis and asthma [165,166]. Regarding MS, a pilot trial involving 17 people with relapsing-remitting MS found that a modified fasting regimen induced changes reminiscent of those seen in rodent EAE models, including similar, possibly beneficial alterations to the gut microbiota [163]. The FMD may also improve the clinical and quality of life scores in people with relapsing-remitting MS [164].

## 4. Challenges to Implementing Fasting in Neurological Disease

To properly apply fasting as a therapy in neurological disease, it is essential to recognize when fasting may or may not be indicated, know how to manage common adverse effects that may occur, and be aware of several common misconceptions.

### 4.1. Potential Contraindications and Adverse Effects of Fasting

Not all individuals are suitable for fasting, and even the most suitable candidates may develop fasting-related adverse effects (Table 3). Most adverse effects can be avoided by ensuring adequate fluid and salt intake combined with a good balance between exercise and rest [65].

#### 4.1.1. Potential Contraindications

Studies involving fasting regimens in people of below-normal body weight, breastfeeding or pregnant women, children, and the very old have been relatively scarce; in these people, fasting should be initiated cautiously, or not at all. Individuals highly susceptible to malnutrition are not suitable for a fasting regimen, including those with a neurological disease; for example, fasting is contraindicated in certain people with PD or AD who may be malnourished [139]. Though the role of fasting in acute infections has not been fully elucidated in humans, fasting may be detrimental in viral infections (conversely, it may be protective in bacterial infections) [167]. Fasting can still be considered in individuals with type 1 or 2 diabetes, gastroesophageal reflux, renal stones, and gout; however, it would be wise to first consult a physician experienced in fasting. Given the evidence that fasting can improve or reverse insulin resistance [66,87,88,89], people with type 2 diabetes are usually ideal candidates. Moreover, the risk of fasting-induced hypoglycemia in type 2 diabetes is low; a recent study examining the effects of type 2 diabetics adhering to a two-days-per-week fasting regimen over 12 weeks demonstrated that most participants did not experience hypoglycemia (defined as a blood glucose level < 4.0 mmol/L), and no participant experienced severe hypoglycemia (defined as an event requiring the assistance of another person for its correction) [168].

#### 4.1.2. Common Adverse Effects

In a recent, comprehensive analysis of 768 visits involving individuals maintained on a medically supervised, water-only fast for two or more days, most adverse effects were mild-to-moderate and included (in descending order) fatigue, insomnia, nausea, headache, hypertension (deemed incidental, given that 97% of people with hypertension as an “adverse effect” also had hypertension as their dominant medical complaint), presyncope, dyspepsia, back pain, and pain in an extremity [169]. It has long been known that the initial days of a period fasting are associated with a natural diuresis, or “natriuresis of fasting,” in which large amounts of water and sodium are lost in the urine [20,170]. In fasting periods lasting 24–48 hours or longer, the natriuresis exposes an individual to dehydration and low sodium levels, which if left untreated, can produce symptoms such as fatigue, headache, and presyncope; in most cases, symptoms related to the natriuresis can be avoided by ensuring adequate water and salt intake.

#### 4.1.3. Rare Adverse Effects

Extremely rare adverse effects have been documented in individuals undergoing prolonged fasts, including edema, severe hypokalemia, bowel obstruction, urate nephrolithiasis, ventricular arrythmias, and even death [20,63,171,172]; however, it is essential to recognize that all of these adverse events have occurred in people undergoing extremely prolonged fasting periods, many lasting several weeks or months in duration. In contrast, out of 768 visits involving individuals undergoing water-only fasting for two or more days, none of these rare adverse effects occurred [169].

### 4.2. Misconceptions of Fasting

Sometimes, confusion arises regarding the potential effects of fasting in humans. Usually, an understanding of physiological context allows any misconceptions to be clarified.

#### 4.2.1. Symptomatic and Metabolic Effects of Fasting Versus Severe Calorie Restriction

It is important to differentiate the symptomatic and metabolic effects of a virtual elimination of calories (fasting) from those associated with a severe, 40%–50% calorie restriction. Individuals undergoing short-term fasts frequently report a lack of hunger, which may be proportional to the level of ketosis achieved, as well as improvements in energy, mood, self-confidence, and quality of life [55,56,88,93,166,173]. In contrast, severe calorie restriction is associated with persistent hunger, fatigue, irritability, apathy, and loss of sex drive [174]. These contrasting symptomatic effects may result from documented differences between severe calorie restriction and fasting with respect to their effects on the resting metabolic rate. The human body adapts to a chronic 20%–40% reduction in calorie intake by lowering its resting metabolic rate to roughly the same degree [174,175]. In contrast, fasting stimulates a 5%–15% increase in the resting metabolic rate, which generally peaks two-to-three days after the initiation of the fasting period [176,177,178,179], after which the metabolic rate lowers to more or less its original rate [180,181,182]. The underlying mechanisms for these contrasting metabolic responses are largely explained by the fact that calorie restriction reduces overall sympathetic activity, whereas fasting increases it via the activation of “counter-regulatory” hormones such as GH, cortisol, and catecholamines [39,178,183,184].

#### 4.2.2. Muscle Mass and Exercise Tolerance

The effects of fasting on muscle mass and exercise tolerance are frequently debated. In any individual, the degree of weight loss, including muscle loss, depends on their initial body fat, calorie and protein intake, and exercise levels [185]. In overweight and obese individuals, protein intakes of 0.8–1.2 g per kg of body weight per day have a sparing effect on lean mass [186]. However, low and normal weight individuals display higher rates of protein oxidation relative to energy expenditure compared to obese individuals [179], suggesting that leaner people may require more protein per kg of body weight to maintain muscle mass. In addition to adequate protein intake, regular exercise has also been shown to prevent muscle loss in obese and normal weight individuals undergoing fasting regimens. In a 12-week study involving obese individuals, combining ADF with endurance exercise three times per week reduced fat mass and retained lean mass in a superior manner to either ADF or exercise alone [187]. Moreover, two recent studies involving healthy young men showed that TRF combined with resistance exercise three times per week resulted in decreased fat mass and energy intake, whereas lean mass and strength were retained [188,189]. These findings suggest that exercise is not significantly limited by fasting; moreover, exercising in the fasted state may actually be an ideal method for decreasing fat mass while retaining muscle.

#### 4.2.3. Fasting-Induced Insulin Resistance

It has long been recognized that fasting periods exceeding 48 hours in humans are often accompanied by a decrease in skeletal muscle insulin sensitivity [38,190]. This fasting-induced insulin resistance, also known as “starvation diabetes,” develops in the setting of hypoglycemia and hypoinsulinemia and probably serves to limit glucose uptake by skeletal muscle, ensuring that a steady glucose supply always remains available for the obligatory requirements of the brain [190]. Thus, fasting-induced insulin resistance represents a normal physiological adaptation that aims to preserve brain function. It is important to distinguish fasting-induced insulin resistance from insulin resistance that develops in the setting of hyperglycemia and hyperinsulinemia, since the latter is pathological and may lead to type 2 diabetes

#### 4.2.4. Compensatory Overeating

A final concern is that at the end of each fasting period, individuals may become susceptible to compensatory overeating, an effect that would mitigate the beneficial effects of the fast. For over a century, increased hunger leading to “post-restriction hyperphagia” has been documented in people subjected to severe calorie restriction regimens [174,175,191,192]. In contrast, recent studies of people on fasting regimens have not shown compensatory overeating on feeding days [55,56]. Moreover, in studies that have reported an increase in calorie intake on the feeding days, the extra intake has still not compensated for the overall calorie deficit induced by the fasting periods [193].

## 5. Conclusions

In an era of rising healthcare costs and an increasing prevalence of neurological disease, the introduction of a self-empowering, cost-free, effective therapeutic option for a range of neurological disorders would be a welcome addition to the armamentarium of physicians. Today’s most common neurological disorders are fundamentally characterized by defective metabolism, on many levels. Given that fasting is a simple, multi-targeted, and essentially “metabolic” therapy with a healthy track record for treating a variety of neurological diseases in animals, it holds promise as a treatment for analogous diseases in humans. Despite this promise, the state of the evidence in humans is extremely limited; many more studies are needed before the actual clinical efficacy of fasting as a therapy in human neurological disorders can be ascertained. Yet if these studies can be prioritized, perhaps the day will come when fasting regimens are prescribed alongside medication-based approaches, culminating in the inception of a unified metabolic approach, capable of modifying not only the symptoms, but also the natural course, of the most common, disabling neurological diseases in existence.

## Figures and Tables

**Table 1:** Human fasting regimens (by intensity, frequency, and duration).

Intensity of Food and Drink Restriction	Frequency and Duration of Fasting Periods	Common Combinations Used in Human Studies
“Pure” fasting (no food or drink, often in the context of religious practices)	Time-restricted feeding (daily four-to-twelve hour eating window)	Water/fluid-only time-restricted feeding
Water-only fasting (only water is permitted, plus salt and micronutrients)	Alternate-daily fasting (fasting every other day)	Water/fluid-only alternate-daily fasting
Fluid-only fasting (water-only fast plus calorie-free fluids, such as tea and black coffee)	Two-days-per-week fasting (fasting two consecutive days per week)	Limited calorie intake two-days-per-week fasting
Limited calorie intake fasting (up to 250–500 kcal per day, via vegetable or bone broths)	Periodic fasting (fasting periods two days to three weeks in duration)	Limited calorie intake periodic fasting

**Table 2:** Summary of evidence for fasting as a therapy in the prevention and treatment of common neurological diseases (in both animals and humans).

Disorder	Evidence in Animals	Evidence in Humans
Metabolic syndrome (a major risk factor for neurological disease)	Mitigates obesity Improves insulin sensitivity Alleviates hypertension	Mitigates obesity Improves insulin sensitivity Alleviates hypertension
Cancer (including cancers that involve the brain)	Probably prevents formation of tumours, and possibly treats established tumours Improves tumour responses to chemotherapy	Ameliorates chemotherapy-related adverse effects May protect normal cells from chemotherapy
Neurodegeneration	Improves cognition, and stalls age-related cognitive decline Usually slows neurodegeneration	No direct evidence (only indirect evidence of benefit from ketogenic diets)
Stroke	Reduces brain damage Enhances functional recovery	No direct evidence
Epilepsy	Probably lessens severity and frequency of seizures	Lessens severity and frequency of seizures
Multiple sclerosis	Mitigates pathology and symptoms of experimental autoimmune encephalomyelitis	No direct evidence (only indirect preliminary evidence of benefit from fasting-mimicking diets)

**Table 3:** Potential contraindications and common adverse effects of fasting.

Potential Contraindications	Common Adverse Effects
People of low body weight	Fatigue
Breastfeeding or pregnant women	Insomnia
Extremes of age (children, the very old)	Nausea
People at high risk of malnutrition	Headache
Viral infections	Presyncope
Type 1 diabetes	Dyspepsia
Renal stones	Back pain
Gout	Pain in extremity
